# Design, Fabrication and Testing of a High-Sensitive Fibre Sensor for Tip Clearance Measurements

**DOI:** 10.3390/s18082610

**Published:** 2018-08-09

**Authors:** Gaizka Durana, Josu Amorebieta, Ruben Fernandez, Josu Beloki, Eneko Arrospide, Iker Garcia, Joseba Zubia

**Affiliations:** 1 Communications Engineering Department, University of the Basque Country (UPV/EHU), Ingeniero Torres Quevedo Plaza 1, E-48013 Bilbao, Spain; josu.amorebieta@ehu.eus (J.A.); ruben.fernandez@ehu.eus (R.F.); ikergeb@gmail.com (I.G.); joseba.zubia@ehu.eus (J.Z.); 2 Aeronautical Technologies Centre, Bizkaia Technological Park, E-48170 Zamudio, Spain; josu.beloki@ctabef.com; 3 Applied Mathematics Department, University of the Basque Country (UPV/EHU), Ingeniero Torres Quevedo Plaza 1, E-48013 Bilbao, Spain; eneko.arrospide@ehu.eus

**Keywords:** tip clearance, optical fibre sensor, aircraft turbine

## Abstract

A highly sensitive fibre bundle-based reflective optical sensor has been designed and fabricated for Tip Clearance measurements in a turbine rig. The sensor offers high spatial and temporal resolution. The sensor probe consists of a single-mode transmitting fibre and two concentric rings of receiving multimode fibres that collect reflected light in a differential detection gain configuration, yielding a highly linear calibration curve for distance measurements. The clearance measurement range is approximately 2 mm around the central point fixed at 3.2 mm from the probe tip, and the sensitivity of the probe is 61.73 mm^−1^. The fibre bundle has been designed to ensure that the distance security specifications required for the experimental program of the turbine are met. The optical sensor has operated under demanding conditions set by the blade and casing design. The experimental results obtained so far are promising and lead us to think that the optical sensor has great potential for online clearance measurements with high precision.

## 1. Introduction

In aeronautics, Tip Clearance (TC) refers to the gap existing between the blade tip and its surrounding case. Since the invention of the gas turbine engine, intense research has been conducted on reducing TC, as this parameter, of great concern for engine designers, is intimately related to engine efficiency and represents the driving force of most new architectures and innovative technological improvements for future aircraft applications. Whereas high TC values allow an amount of air to flow without generating useful work, a lack of clearance accelerates blade tip and shroud wear over time due mainly to rubs, and can put engine integrity at risk [1]. The clearance varies with the operation point of the mission profile (take-off, cruise and landing) as well as with the engine aging [2,3]. TC changes are caused by two types of loads, namely engine and flight loads. The former encompasses centrifugal, thermal, internal engine pressure, and thrust loads, whereas the latter comprises inertial (gravitational), aerodynamic (external pressure) and gyroscopic loads [4]. At cruise, a rule of thumb equates 0.25 mm of reduced clearance to a reduction of 1% in specific fuel consumption. Therefore, some of the most relevant benefits of reducing the TC include efficiency increase as well as increased payload and mission range capabilities [1,5]. In addition, aircraft noise and emissions are reduced, along with the subsequent environmental benefits involved [6,7]. It seems obvious that an accurate and real-time measurement technology is necessary. In contrast to power-system turbines where common clearance values range from 2 to 8 mm, in aircraft turbines, TC is typically lower than 3 mm and a resolution better than 25 µm is usually required [8,9,10].

Currently, there are several traditional methods for TC measurements that include capacitive, eddy current, microwave, and discharging probe sensors (electromechanical). The former are popular due to their simplicity, low cost, and robustness, but they suffer from low spatial resolution, short measurement range, and require calibration [11,12,13]. Eddy current sensing is also a common technique for TC measurements, and it has about the same accuracy as capacitive probes [14,15]. It provides non-contact measurements at the expense of requiring magnetic materials for the blades. Additionally, the magnetic disturbance of the turbine engine may interfere with their output signal, and they are highly sensitive to temperature and blade tip shape. Microwave sensors are robust and insensitive to contamination, but the hollow waveguides at submillimeter wavelengths are impractical, and the corresponding circuitry complex [16,17]. Finally, electromechanical systems belong to the oldest tip clearance measurement systems [18]. They benefit from their high resolution over the entire measurement range, but their main drawbacks are that they only measure the clearance of the longest blade and the slow response time.

Optical sensors may overcome many of the previous drawbacks as they offer high sensitivity and resolution, immunity to electromagnetic interference, non-contact measurement and information about every blade [19,20,21,22]. However, among the different optical technologies (i.e., triangulation [23,24], OCT [22], time-of-flight measurements [25], laser Doppler velocimetry [26] and reflective intensity modulation [27]) employed for TC measurements at turbo machines, many of them do not fully satisfy the requirements of future closed-loop Active Clearance Control (ACC) systems [5]. For example, laser Doppler position probes offer high resolution, but the complexity of the probe limits the application of the system. In the case of the Optical Coherence Tomography (OCT), the measurement rate is limited by the speed of mechanical scanning, or in the case of triangulation, by the detector frame rate and minimum exposure time. Finally, the resolution of reflective intensity modulation-based sensor probes—compared to the rest of optical methods mentioned previously—is low due to the modal noise at the endface of the transmitting fibre.

In this paper, we report on the design and fabrication of a highly sensitive fibre bundle-based reflective optical sensor that has been tested in an aircraft turbine rig. The content of the paper has been structured as follows: first, a brief description of the operation principle is given, explaining the general sensor design and defining the working region of interest that will allow to maximize the sensitivity of the sensor. Afterwards the experimental program followed at the Aeronautical Technologies centre’s (CTA’s) transonic wind tunnel is explicated. Then, the most relevant results are presented and discussed. Finally, some conclusions are drawn from the previous discussion.

## 2. Materials and Methods

### 2.1. Sensor Design and Working Region of Interest

**Figure 1:**
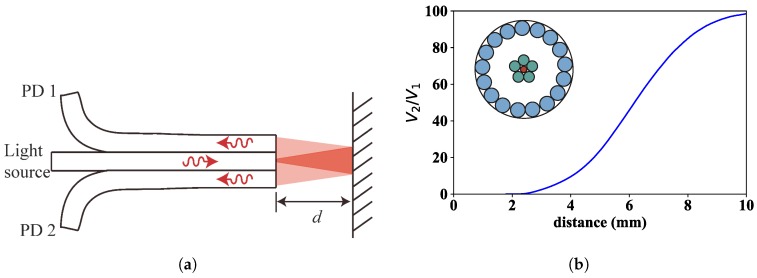
Fibre optic intensity sensor for TC measurements: (**a**) illustration of the fibre bundle based sensor; and (**b**) signal response ($V_{2}/V_{1}$) as a function of the distance *d* to a mirror. The drawing included in the plot shows the cross-section of the fibre bundle, where the single-mode transmitting fibre is in the centre and the two rings of receiving multimode fibres are arranged concentrically around it.

The schematic diagram of the sensor’s operation is shown in Figure 1a. The fibre bundle is the principal element of the system. To avoid modal noise at the output [28], a central single-mode fibre is used as transmitting fibre of red laser light (wavelength of light: 660 nm), which after exiting the fibre bundle and being reflected by the target object located at a distance *d* from the fibre bundle tip, is partially gathered by two concentric rings of multimode optical fibres arranged around the central transmitting fibre; the inner ring consists of 5 optical fibres (fibre bunch 1; core diameters of 200 µm and Numerical Aperture (NA) of 0.2), whereas the outer ring consists of 17 optical fibres (fibre bunch 2; core diameters of 300 µm and NA of 0.2). The light collected by fibre bunches 1 and 2 is measured as a voltage level at photodetectors 1 and 2, respectively ($V_{1}$ and $V_{2}$). If we plot the ratio of $V_{2}$ to $V_{1}$ as a function of distance *d*, we get the characteristic calibration curve shown in Figure 1b. The reason of using two photodetectors is aimed at minimizing the undesirable effects caused by intensity fluctuations in the light source and reflectivity variations on the target surface. As both voltage signals contain the same disturbance, the ratio $V_{2}/V_{1}$ gets rid of it and therefore becomes a pure function of the distance to the illuminated target surface *d* [27,29,30].

The signal response presents two regions of interest for distance sensing with a characteristic linear variation of the signal with distance. Those regions are at both sides of the peak signal and are designated as “front slope” and “back slope”, respectively. Figure 1b only shows the front slope, which exhibits clear advantages in terms of sensitivity, protection against noise and temperature fluctuations, in comparison to the back slope [31]. In practice, however, for distance security reasons typical TC values found in turbine rigs makes it necessary to operate in the back slope of the sensor, resulting in a lower signal sensitivity and higher dependency on the type of surface and on the temperature. Additionally, a post-processing of the raw signal is often necessary to get reliable results [27]. In the present work, we set out to operate in the front slope through the design of a new fibre bundle that guarantees a safe operation without compromising the physical integrity of the sensor head keeping it away from the blades. Indeed, in previous works, we used a fibre bundle with a measurement range for the front slope that clearly was too short (from 1 mm to 1.6 mm), and therefore required using the back slope to avoid placing the fibre bundle tip too close to the blades. The new bundle design (number of fibres, fibre type composition and geometrical fibre arrangement) takes into account all this, and, as a result, is able to shift the front slope to bigger probing distances (4–8 mm, see Figure 1b).

**Figure 2:**
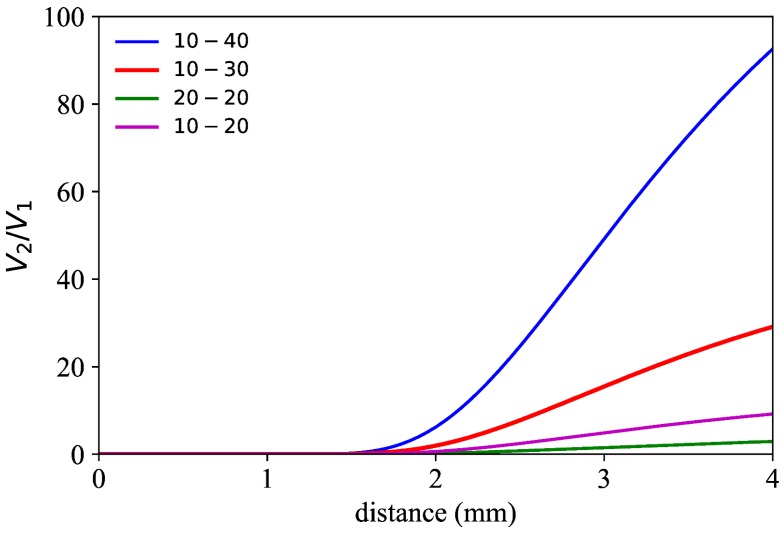
Simulated calibration curves of the fibre bundle in region I for different gain configurations ($G_{1}-G_{2}$) of the two photodetectors.

Regarding the achievable sensitivity in the front slope, differential gain of the photodetectors have been considered to increase it as much as possible provided that the gain configuration of the transimpedance amplifier of each photodetector does not compromise the minimum bandwidth required by the target application. In our particular case, for a turbine with 92 blades spinning at a maximum of 6000 revolutions per minute (rpm), even at the highest gain configuration the bandwidth available is enough to receive a signal with clearly identifiable individual blades. Figure 2 shows simulation results of four different gain configurations $G_{1}-G_{2}$ of the photodetectors ($G_{1}$ for photodetector 1 (PD1) and $G_{2}$ for photodetector 2 (PD2), both given in dB units with respect to the reference gain value of 0.75 × 10^3^ V/A) obtained by a custom designed program for bundle behaviour simulation. As can be clearly observed, the gain increase of the second photodetector with respect to the first one yields not only a higher ratio of $V_{2}$ to $V_{1}$, but also a steeper calibration curve than in the case of the symmetric configuration ($G_{1}=G_{2}$). Therefore, for maximum sensitivity, we have set the gain configuration to 10–40.

### 2.2. Calibration Curve

**Figure 3:**
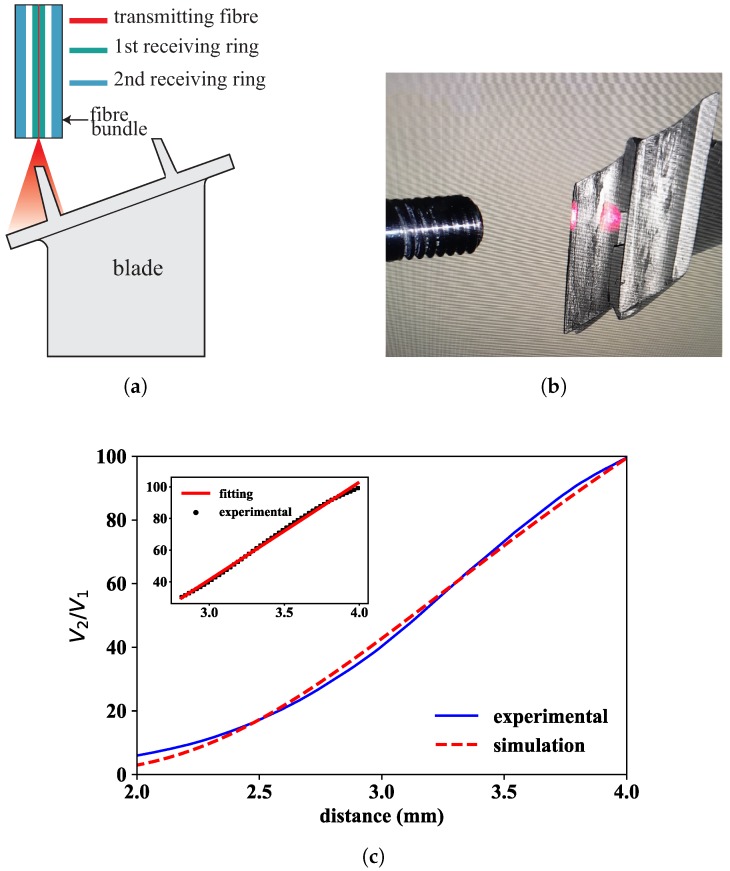
Laboratory calibration of a typical blade from the turbine rig: (**a**) schematic representation of the optical probe tip relative to the spare blade; (**b**) close-up picture of fibre bundle and blade; and (**c**) simulated and measured calibration curves. The inset shows the linear fit to the experimental data in the region of interest (2.8 mm < *z* < 4 mm).

Once the measurement system has been defined, the next step consists in calibrating the optical sensor using as target object a spare blade from the tested turbine rig. The schematic drawing of the side view of the experimental set-up is shown in Figure 3a.

It is important to point out that, in the laboratory calibration tests, the transmitting fibre did not face the flat platform of the blade—as would be desirable to maximize the amount of reflected light gathered back by the bundle—but the very narrow sealing lands of the blade (around 0.7 mm in width) to simulate the real turbine configuration that the sensor head met when installed in the turbine rig. Figure 3b shows a close-up picture of the sensor head during the calibration process. Please note that even perfectly facing the transmitting fibre to the narrow sealing lands yielded a very low reflected signal that required setting the optical power to maximum value, in this case 50 mW. Therefore, the setting of 50 mW laser power and 10–40 gain configuration of the photodetectors always resulted in light intensity levels at each of the photodetectors below the saturation value, and the created voltage values spanned over the full voltage scale (0–5 V), ensuring a good use of the 16-bit resolution of the A/D converter. The large working distance set by the front slope of the fibre bundle also contributed to the low coupling efficiency of reflected light into the fibre bundle. Both simulated and measured calibration curves are shown in Figure 3c.

It is worth mentioning the great similarity between both curves in the distance range from 2 to 4 mm with very small differences between them, and with a clear linear increase of the rate of the voltage quotient with distance. The best linear fit (shown in the inset of Figure 3c) to the experimental data has a Pearson’s correlation of 0.997 in the distance range from 2.8 mm to 4 mm with a sensitivity slope of 61.73 mm^−1^. Within this distance range of interest, Table 1 shows, for different values of $V_{2}/V_{1}$ ranging between 30 and 100, the difference between the experimentally measured distance value and the corresponding value obtained from the simulation. The result is given as a percentage of the corresponding experimental value. The discrepancy between experiment and simulation never exceeds 1.5% (at $V_{2}/V_{1}=34.1$). It is also interesting to draw attention to the shift to lower distance values occurring in the front slope when moving from an object with specular reflection (target presented in Figure 1) to another one with diffuse reflection (blade shown in Figure 3). This can be easily understood with simple geometric and ray tracing models [32].

### 2.3. Experimental Program

The performance of the optical sensor was tested in the transonic wind tunnel at CTA. The rotating-turbine-test facility is a continuous transonic-flow-test bed with an atmospheric inlet/outlet. The level of pressure/vacuum, the temperature and the mass flow are individually regulated, so that the rig is operated to meet realistic Mach and Reynolds numbers allowing to transfer the results to real gas turbines.

**Figure 4:**
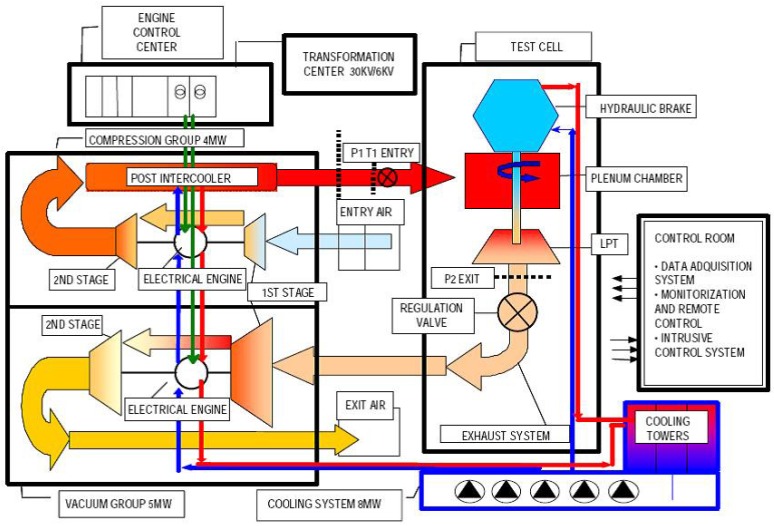
Schematic diagram of the rotating-turbine-test facility at CTA.

The supply and exit air conditions in the test section are achieved by two centrifugal compressor and vacuum groups, which are, respectively, run by electrical motors of 3.7 MW and 5.0 MW. Two vacuum pumps are used to achieve altitude conditions of sub-atmospheric pressure down to 12.5 kPa. A two-stage compressor group is used to control the pressure ratio and flow temperature and thus the Mach number of the flow within the circuit. The top mass flow rate achievable is 18 kg/s, with a maximum supply pressure up to 450 kPa, and a temperature regulation from atmospheric up to 450 K. Prior to entering the turbine, the air flows through a settling chamber that removes any swirl and axial velocity non-uniformity. The turbine power is transmitted by a single shaft (up to 7800 rpm) to a dynamometer. The test section has a section of 1 m. A schematic diagram of the facility is shown in Figure 4.

The rig corresponds to a single stage of a turbine rig with 92 blades. As already commented previously, the measurement requirements were really demanding because, for the extremely narrow sealing lands of the blades that defined the reflecting surface, the distance of about 3.2 mm from where the end of the probe was finally set, to the reflecting surface caused a low reflected signal level at the receiving rings to happen. An additional challenge that posed the coupling of the optical probe to the casing of the turbine was that the optical probe was not perfectly faced to the sealing lands when the turbine was at rest (0 rpm), so that the reflected signal was too low to get reliable calibration data that would allow building the calibration curve for the actual measurements. For that reason, the laboratory calibration curve was accepted as valid for the turbine rig measurements since it was carried out with a spare blade of the same turbine stage under test. As shown in the Results Section, the good news is that, at different workload conditions of the turbine rig, the optical probe was able to receive enough reflected signal for reliable tip clearance measurements. This improvement in the level of reflected signal was a consequence of the several vibrations that tend to suffer rotor blades causing them not only to deform but also to get a better alignment of the reflecting surface with respect to the optical probe.

**Figure 5:**
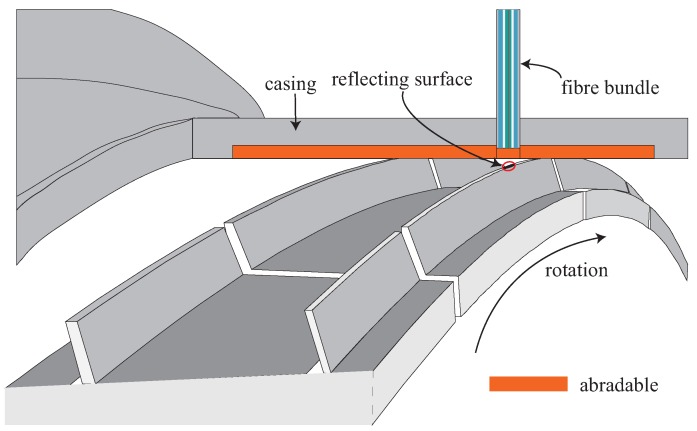
Schematic representation of the optical probe (not to scale) installed in the turbine rig at CTA.

Figure 5 shows a schematic representation of the final arrangement of the bundle embedded in the casing of the turbine. The optical probe was attached to a micrometer-driven adapter that was inserted in a radial hole of the casing and fixed to it with four screws. The micrometer allowed to set a certain distance—3.2 mm in this particular case—between the probe tip and the sealing lands of one of the blades to set the operation point at the middle of the linear region shown in Figure 3c. For this particular configuration, the optical probe tip resulted to be within the abradable layer. The abradable is a soft protective wear material that is mounted on the casing wall aligned with the blades to create a good sealing, and avoid gas leakage and improve combustion efficiency. As TC values are commonly referenced to the abradable coating, below, we consider this case. Therefore, according to turbine design blueprints, the TC is obtained subtracting 2.74 mm to the actual sensor measurement (distance from probe tip to reflecting surface):

(1) TC(mm)=sensormeasurement(mm)−2.74

**Figure 6:**
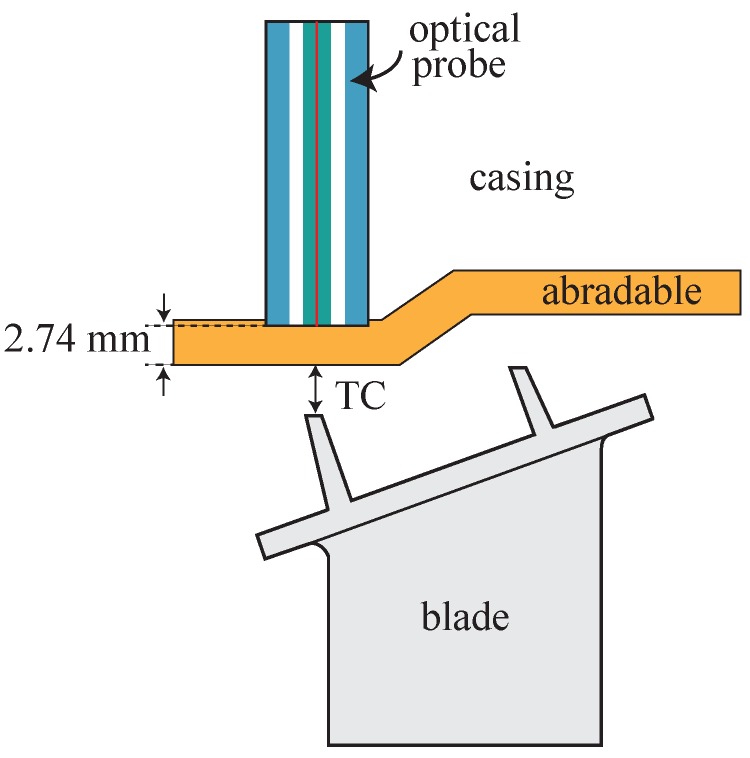
Optical probe placement within the casing.

Figure 6 shows a schematic illustration of the optical probe configuration within the casing. The optical signal of each of the two photodetectors is acquired with 16-bit resolution at a sampling rate of 2 MS/s, which results in a detailed map of all the blades with unambiguous identification of each of them, extending further the information provided by classical electromechanical systems that limit the TC information to the longest blade, and with a much lower data refresh frequency. The data acquisition and processing was done with a custom-made LabVIEW program that allows online and offline working modes. In online mode, the TC values of the different blades are monitored live at a configurable refresh rate, whereas, in offline mode, the data are stored in a hard disk for later processing. The latter mode is particularly interesting for long acquisition times where the amount of data created is huge and a thorough data analysis is required.

## 3. Results and Discussion

**Figure 7:**
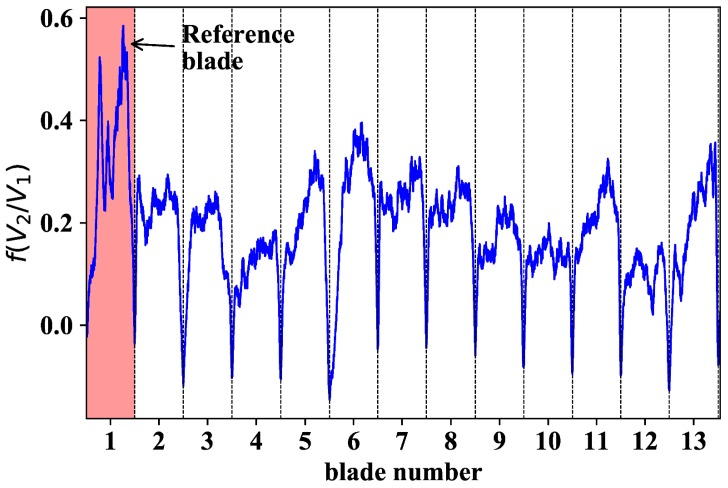
Typical signal response $V_{2}/V_{1}$ after applying the corresponding calibration curve. The blade highlighted in red refers to the blade with a higher reflection pattern. The dashed vertical lines correspond to local minima defining the limit between adjacent blades.

All tests were carried out at CTA’s facilities where different Working Points (WP) of the engine were repeated during several days. Each engine revolution was identified both with a blade of a particular reflection pattern and a stable non-vibrating Once per Revolution (OPR) signal obtained from the shaft. The raw data ($V_{2}/V_{1}$) from the optical sensor were converted to distance value using the linear calibration curve $f(V_{2}/V_{1})$ obtained in the laboratory calibration tests (see Section 2.2). As an example, Figure 7 shows the sensor response of 13 blades after applying the calibration curve without any type of data post-processing. The first feature worth observing is the sharp minima that define the gap between consecutive blades.

**Figure 8:**
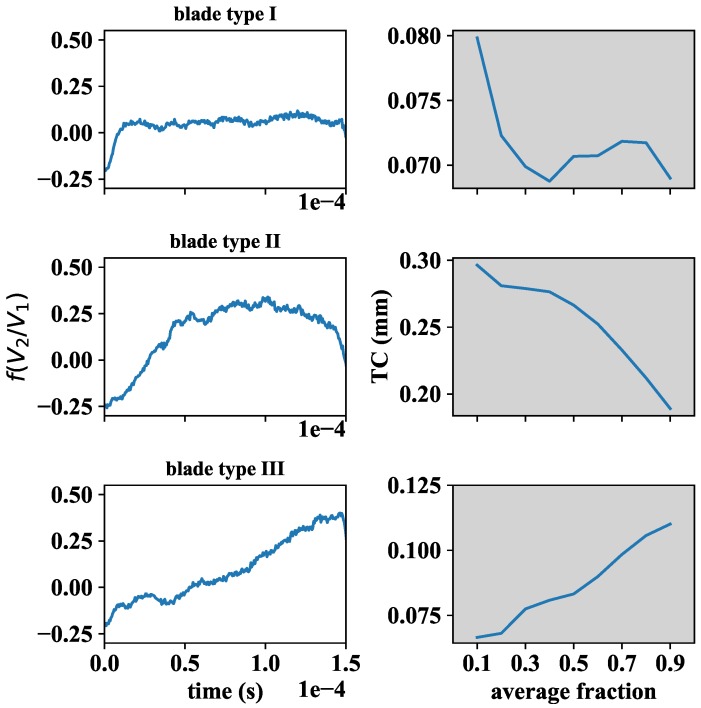
(**Left**) Types of blade signals found in the 92 blade turbine rig after applying the calibration curve; and (**Right**) evolution of TC definition with the selected fraction of blade data for each blade type.

It is also worth mentioning that the signal pattern corresponding to each blade was highly reproducible over time, regardless of the operation point of the engine. The response curve of each of the 92 blades followed a certain pattern that might be classified according to one of the three types presented in Figure 8 (curves shown on the left-hand side of Figure 8). To give a consistent definition of the TC, for each blade, we started selecting a variable percentage (from 0% to 100%) of the corresponding dataset around the central data sample, and analyzed the evolution followed by the average value. The curves on the right-hand side of Figure 8 show the variability of the given TC definition for each of the three blade types. It comes out that, regardless of the type of response curve considered, the average value variation always was below a tenth of a millimetre. Therefore, it was decided to define the TC of each blade as the average value of the corresponding dataset at the 50% selection level around the central sample.

**Figure 9:**
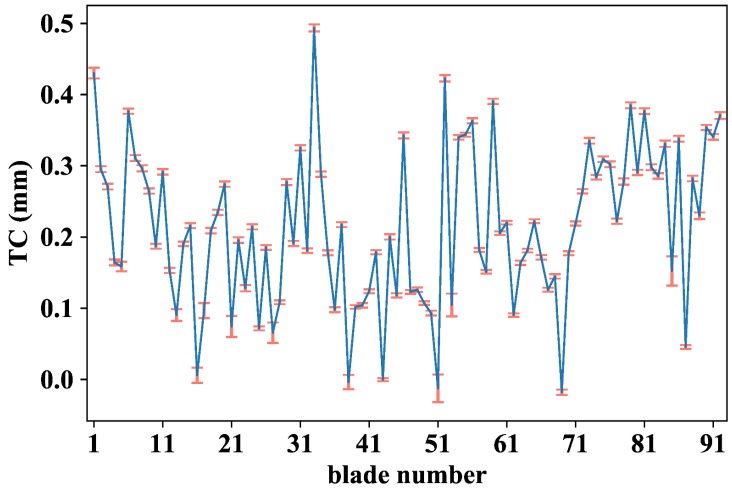
TC values of each blade at 4258 rpm with error bars that account for the TC standard deviation over 1100 revolutions.

With the given definition of TC in mind, Figure 9 shows the TC map corresponding to a certain engine WP at 4258 rpm. It is worth noticing that the TC of every blade is different. Of particular importance are blades number 16, 38, 43, 51 and 69 as their TC values suggest that they are close to the abradable surface and they should be monitored to keep them under control. Regarding the stability of the test, the vertical error bars represent the variability of the TC values over time. Even in the worst case (blade number 85), the TC variability expressed as a single standard deviation value is approximately of 20 µm within the same WP, and the average value over the 92 blades is below 5 µm. All this suggests that we are dealing with stable TC experiments.

**Figure 10:**
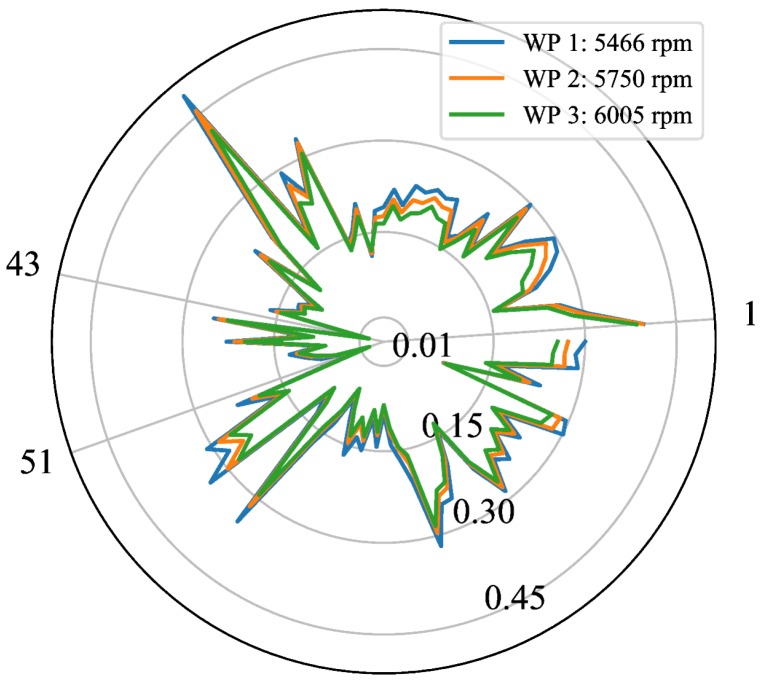
TC values of each of the 92 blades at three different WPs.

The TC maps of all blades corresponding to three different WPs in ascending order of rpm are shown in the polar plot of Figure 10. Each TC map is represented as a curve of a particular color. On the other hand, each blade is expressed as a single point where the polar angle $\theta_{i}$ defines the blade number *i*—$\theta_{i}=360^{\circ }/92*i$ for i∈[1,92]—and the corresponding TC value is given by the radial distance. Observe that TC values of individual blades decrease as engine rotational speed increases, a fact that can be attributed to the centrifugal and thermal loads acting on static and rotating components of the turbine. It is also interesting to point out that blades 43 and 51 still continue to be decisive in determining the TC values of the turbine, in the same way as in the case of the WP at 4258 rpm shown in Figure 9. If we define the turbine TC as the minimum blade TC among all blades, as expected, the TC decreases from 0.002 mm to −0.005 mm when going from WP 1 (5466 rpm) to WP 3 (6005 rpm).

**Figure 11:**
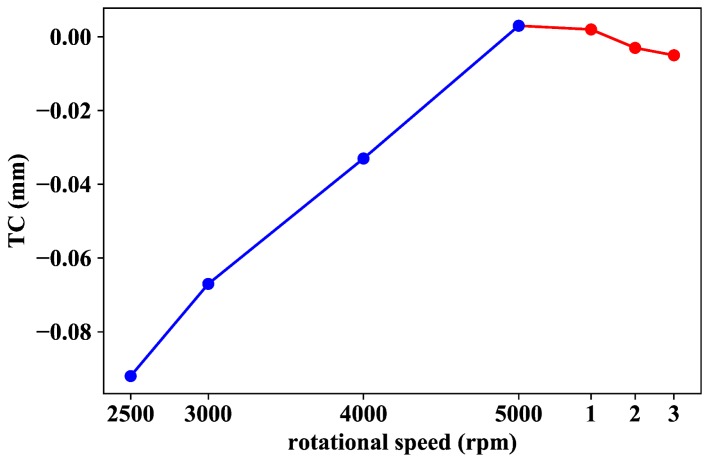
TC behaviour before (blue data points) and after (red data points) setting the first WP. The red data points refer to WPs 1, 2 and 3 shown in Figure 10: WP 1 → 5466 rpm; WP 2 → 5750 rpm; and WP 3 → 6005 rpm.

Another interesting point to consider is the analysis of the TC evolution when the turbine rpms ramp up before arriving at the first WP. Blue data points shown in Figure 11 are representative of this case. Contrary to what is expected for the general case in which the TC diminishes when rotor speed increases (as already shown in Figure 10), during the warming-up lapse of time, the clearance increases with rpm values. This might be explained on the basis that, when speed increases, the centrifugal load of the rotor as well as the rapid heating of the blades cause the rotating elements to grow outwards, but the case expands at a faster rate during this process.

This observation brings us to conclude that temperature ramping occurring in the wind tunnel before the first operation point is reached might be associated with the observed TC increase with rotational speed. In addition, the blade-case rubbing experienced during the whole warming-up process (negative TC values of blue data points in Figure 11) may be justified if we consider that the centrifugal load on the blades applies from the first moment before the casing starts to expand. However, once the operation temperature has been reached and the casing has expanded to its equilibrium value (first red data point in Figure 11: WP 1 → 5466 rpm), the clearance starts to decrease with rotational speed as expected.

## 4. Conclusions

A highly sensitive optical fibre bundle-based sensor prototype was designed and fabricated based on a custom simulation program developed within the research group. The manufactured optical sensor probe allowed measuring TC values in a turbine rig of an aircraft engine at the wind tunnel of the CTA. The optical measurements rely on collecting reflected light from each of the blades using two concentrically arranged rings of optical fibres and converting the gathered light intensities into voltage levels that are eventually divided with respect to each other to get rid of the disturbances (light intensity fluctuations, reflectivity variations, etc.) and retain a pure function of the distance from the fibre bundle tip to each blade. This curve has two characteristic working regions of interest with linear behaviour, the so-called front slope and back slope. The added value of the present work with respect to previous works resides in shifting the highly sensitive front slope curve to longer distance values to meet the distance security specifications set for the experimental program of the turbine, a fact that enables establishing the working point around the central part of this sharply sloped curve section instead of using the less sensitive back slope section of the response function. Additionally, the sensitivity has been further improved using differential gain of the two photodetectors associated to their corresponding receiving fibre rings. Altogether, the sensitivity of the optical sensor is 61.73 mm^−1^, in contrast to the value of −0.0733 mm^−1^ published by other authors [33]. It is also worth mentioning that the optical sensor has proved to be capable of measuring the TC in very unfavourable conditions set by the specific blade and casing design that prevented the sensor from receiving an appreciable level of reflected signal. In such demanding scenario, the calibration curve used for the actual measurements was obtained in the laboratory using a spare blade of the turbine as it was impossible to get reliable calibration data from the turbine at rest. The results derived from the experimental program carried out on a turbine rig at CTA’s facilities show a high resolution and highly sensitive measurement tool for inspection of individual blades that provides engineers with valuable information on turbine performance. The results of the optical fibre-based sensor presented in this paper opens up the possibility of widening its applicability to other fields of interest.

## Figures and Tables

**Table 1:** Comparison of measured and simulated data in the distance region of interest.

$V_{2}/V_{1}$	$d_{\mathrm{sim}}$, mm	$d_{\exp}$, mm	Difference, %
30.329	2.778	2.815	1.31
45.930	3.055	3.090	1.13
59.443	3.289	3.290	0.11
75.959	3.543	3.540	0.83
90.551	3.831	3.790	1.08
99.171	3.992	3.990	0.06
